# Spatial distribution and associated factors of underweight in Ethiopia: An analysis of Ethiopian demographic and health survey, 2016

**DOI:** 10.1371/journal.pone.0242744

**Published:** 2020-12-01

**Authors:** Biruk Shalmeno Tusa, Adisu Birhanu Weldesenbet, Sewnet Adem Kebede

**Affiliations:** 1 Department of Epidemiology and Biostatistics, College of Health and Medical Sciences, Haramaya University, Haramaya, Ethiopia; 2 Department of Epidemiology and Biostatistics, Institute of Public Health, College of Medicine and Health Sciences, University of Gondar, Gondar, Ethiopia; University of Western Australia, AUSTRALIA

## Abstract

**Background:**

Underweight is one form of indicators of under-nutrition, which results from the poor nutrient intake and underlying health problems. Its impact is beyond an individual and extends to a country level. It has been known from the literature that underweight has a negative effect on income and development of a country. In the context of Ethiopia, factors predicting underweight remain unknown and there is a paucity of evidence on geographical distribution of underweight among individuals aged 15–49 years. Therefore, the aim of this study was to examine the geographic distribution of underweight and its associated factors among individuals aged 15–49 years in Ethiopia.

**Methods:**

Secondary data analysis was done on a data set consisting of 28,450 individuals and obtained from the Ethiopian Demography and Health Survey (EDHS) 2016. The spatial distribution of underweight across the country was identified by ArcGIS software. Hotspots analysis was done using Getis-Ord Gi* statistic within ArcGIS. In SaTScan software, the Bernoulli model was fitted by Kulldorff’s methods to identify the purely spatial clusters of underweight. A binary logistic regression was applied to determine factors associated with being underweight.

**Result:**

In Ethiopia, the spatial distribution of underweight was clustered with Global Moran’s I  =  0.79 at p-value < 0.0001. The highest underweight clusters were observed in Tigray, Gambella, eastern part of Amhara, and western and central part of Afar regions. Male individuals [AOR = 1.21; 95% CI: (1.15 1.28)], never married [AOR = 1.14; 95% CI: (1.05, 1.24)], rural residents [AOR = 1.32; 95% CI: (1.18, 1.47)], rich [AOR = 0.85; 95% CI: (0.76, 0.94)], cigarette smoking [AOR = 1.25; 95% CI: (1.07, 1.46)], drinking treated water [AOR = 0.91; 95% CI: (0.83, 0.99)] and open filed defecation [AOR = 1.17; 95% CI: (1.08, 1.26)] were found to have a significant association with being underweight.

**Conclusions:**

There was a significant clustering of underweight among individuals aged 15–49 years. Gender, age, marital status, place of residence, wealth index, cigarette smoking, using untreated water and types of toilet were the significant factors of being underweight. Therefore, effective public health interventions like building safe and supportive environments for nutrition, providing socio-economic protection and nutrition-related education for poor and rural resident would be better to mitigate these situations and associated risk factors in hot spot areas. In addition, policymakers should strengthen and promote nutrition sensitive policies and activities in order to alleviate the underlying and basic causes of underweight.

## Background

Underweight is a one form of under-nutrition, defined by the World Health Organization (WHO) as body mass index (BMI) less than 18.5 kg/m^2^ [1]. It also refers to deficiencies, in a person’s intake of energy and/or nutrients that leads to low productivity among adults and it is related to heightened morbidity and mortality [2]. Underweight results from poor nutrient intake and underlying health problems. Patients with such chronic diseases as HIV/AIDS, respiratory infections, malaria and diarrheal disease are prone to being underweight [3].

The prevalence of underweight is low in developed countries, but it remains high in developing countries. Globally, 462 million adults are underweight and more than one third of Low and Middle Income Countries face double burdens of both under and overweight, particularly in sub-Saharan Africa, South Asia, and East Asia and the Pacific [4,5]. According to regional WHO reports, South East Asia and Africa have the highest prevalence of underweight among adults, with 20.3% and 11.1% of adults in the region being underweight, respectively. In Ethiopia, the prevalence of adult underweight is 17.2% among men and 14.5% among women with an overall prevalence of 15.8% [6].

Compared to people of normal-weight, Underweight people have increased risk of mortality [7]. Researchers found that Having BMI less than or equal to 22.5kg/m2 increased the risk of death by 1.48-fold in women and 1.26-fold in men [8–10]. Moreover, chronic malnutrition in women of childbearing age is also linked to poor pregnancy outcomes [11,12]. Malnourished mothers are more likely to give birth to babies with low birth weights who are at a greatly increased risk of death in infancy. Women who are underweight prior to pregnancy and gain little weight during pregnancy are at increased risk of complications and death [13,14].

The effect of underweight extends beyond an individual and it has a negative impact on income and development of a country [15]. It is a particular concern for developing countries as it is a leading cause of death and disability [16]. Lower level of education, low income, inadequate health care coverage, food habits and a sedentary lifestyle are factors that are responsible for high burden of underweight in developing countries [17,18].

In order to manage the negative consequences of underweight and to mitigate the problems associated with it, exploration of underlying socio-economic factors is crucial. Despite its high prevalence, factors influencing underweight remain unclear in Ethiopia. In addition, there is a paucity of evidence on geographical distribution of underweight among individuals aged 15–49 years in the country. Therefore, the current study applies spatial analysis and logistic regression to determine the geographic distribution of underweight and its associated factors among individuals aged 15–49 years in Ethiopia.

## Methods

### Study setting and data source

Ethiopia is located in the horn of Africa and has nine regions (Afar, Tigray, Amhara, Oromia, Somali, Southern Nations, Nationalities, and People’s Region (SNNPR), Benishangul Gumuz, Gambella and Harari) and two administrative cities (Addis Ababa and Dire Dawa) [15]. The current population of Ethiopia is 114,864,753 as of Monday, June 22, 2020, based on Worldometer elaboration of the latest United Nations data [16].

The data for this study was taken from the demography heath survey (DHS) program official database www.measuredhs.com, after authorization was granted through online request by explaining the goal of our study. The 2016 EDHS was the fourth Demographic and Health Survey conducted in Ethiopia from January 18, 2016, to June 27, 2016 [19].

The 2016 EDHS used a two-stage stratified cluster sampling method. In the first stage, a total of 645 Enumeration Areas (EAs) (202 in urban areas and 443 in rural areas) were selected with probability proportional to EA size and with independent selection in each sampling stratum.

In the second stage of selection, a fixed number of 28 households per cluster were selected with an equal probability systematic selection from the newly created household listing [19]. The 2016 EDHS collected anthropometric data on height and weight for both women and men age 15–49 years. In the present study, total weighted samples of 28,450 individuals were included. Latitude and longitude coordinates had been also taken from selected EAs (clusters). The specific sampling system was presented in the full EDHS report [19].

### Outcome and explanatory variable

The outcome variable for this study was BMI, which is categorized as underweight if BMI is less than 18.5 and non-underweight if the BMI is greater than or equal to 18.5. Depending on different literature review; independent variables included in the analysis are described in **Table 1**.

**Table 1:** Description of explanatory variables used in the analysis.

Variables	Description	Category
Gender	Sex of household member	Female, Male
Age (years)	Age of household member	15–19, 20–24, 25–29, 30–34, 35–39, 40–44, 45–49, 50–54 and 55–59
Marital status	Never married, Currently married & Formerly married (It includes divorced, separated and widowed)	Never married, Currently married & Formerly/ever married
Educational level	Highest educational level attained	No education, Primary, Secondary, Higher and Don’t know
Number of household members	Number of household members	1–3, 4–6 and >6
Place of residence	Type of place of residence	Urban and Rural
Region	Region	Tigray, Afar, Amhara, Oromia, Somali, Benishangul, SNNPR, Gambella, Harari, Addis Ababa and Dire Dawa
Wealth index	It is the percent distribution of the de jure population by wealth quintiles and the Gini coefficient.	Poorest, Poorer, Middle, Richer & Richest
Drinking water treatment	Anything done to water to make safe to drink	Yes, No and Don't know
Types of toilet	Types of toilet facility	Flush, Pit latrine, Open field and Other*
Cigarette smoking	Smoking cigarettes in last 24 hours	Yes and No

*Other: Composting toilet, bucket toilet, hanging toilet/latrine; SNNPR: Southern Nation and Nationality and Peoples Region.

### Data processing

We used STATA 14; ArcGIS 10.1 and SaTScan 9.6 software’s to perform data analysis. Before any statistical analysis, the data was weighted using sampling weight (household sample weight), primary sampling unit, and strata to restore the representativeness of the survey and to tell the STATA to consider the sampling design when calculating standard errors to get reliable statistical estimates. Descriptive statistics and summary statistics were presented using text and tables.

### Spatial analysis

#### Spatial autocorrelation analysis

Global Moran’s index (Moran’s I) was used to identify the presence of spatial autocorrelation. Moran’s I values close to -1 indicated disease/event dispersed, whereas Moran’s I values close to + 1 indicated disease/event clustered, and disease/event distributed randomly if Moran’s I value was zero. A statistically significant Moran’s I (p < 0.05) led to the rejection of the null hypothesis (underweight is randomly distributed) and indicated the presence of spatial autocorrelation. Hotspot analysis was done using as Getis-Ord Gi* statistic.

#### Spatial scan statistical analysis

Statistically significant Primary (most likely) and secondary clusters of underweight was identified by applying Spatial scan statistics using Kulldorff’s SaTScan software. SaTScan™ works with a moving window and requires fixing of the window size that moves across the study area. The outcome variable has a Bernoulli distribution, so Bernoulli model was used by applying the Kulldorff’s method for purely spatial analysis.

Individuals who were underweight were taken as cases and those who were not underweight were taken as controls to fit the Bernoulli model. The default maximum spatial cluster size of < 50% of the population was used as an upper limit, which allowed both small and large clusters to be detected and ignored clusters that contained more than the maximum limit. Areas with high Log Likelihood Ratio and significant p-value were considered as areas with high underweight compared to areas outside of the window.

### Statistical analysis

In EDHS data, individuals within a cluster may be more similar to each other than individuals in other cluster. This violates the assumption independence of observations and equal variance across clusters. This implies that the need to consider the between-cluster variability using advanced models. Since the response variable was dichotomous, binary logistic regression and Generalized Linear Mixed Model were fitted. The Intra-class Correlation Coefficient (ICC) value was 0.036 which indicated us to select a fixed model (binary logistic regression) over the mixed model.

Variables with p-values ≤0.2 in the bi-variable analysis were fitted in the multivariable model to measure the effect of each variable after adjusting for the effect of other variables. Adjusted Odds Ratio (AOR) with a 95% Confidence Interval (CI) and p-value < 0.05 in the multivariable model were declared as determinant factors of underweight. Multi-collinearity and model adequacy were also checked using a variance inflation factor (VIF) and Hosmer-Lemeshow test respectively.

### Ethics approval and consent to participate

Permission for data access was obtained from major demographic and health survey through the online request from http://www.dhsprogram.com. The data used for this study were publicly available with no personal identifier. They gave permission to access the data with reference number of 144044.

## Results

### Characteristics of study population

A total of weighted sample of 27,214 individuals were included in the analysis. Among these study participants, more than half (53.35%) of them were females, more than one-third (36.84%) of them were not educated, more than three-fourth (79.57%) were rural dwellers and one-quarter (25.39%) of them were richest. About 6,052 (22.24%) of the study participants were in the age range of 15–19 years. Regarding the marital status, around 16,189 (59.49%) respondents were married, whereas 9,075 (33.35%) respondents were never married. Nearly two-third (65.18%) of the study participants had pit types of latrine and only 9.16 percent of respondents had done something to the water to make safe to drink **(Table 2).**

**Table 2:** Characteristics of respondents in Ethiopia from January 18 to June 27, 2016.

Variables	Un-weighted frequency	Weighted frequency	Weighted percentage
**Gender**			
Female	14,099	14,529	53.35
Male	11,285	12,695	46.65
**Age**			
15–19	5,707	6,052	22.24
20–24	4,351	4,439	16.31
25–29	4,510	4,797	17.62
30–34	3,464	3,903	14.34
35–39	3,149	3,409	12.53
40–44	2,374	2,545	9.35
45–49	1,829	2,069	7.60
**Marital status**			
Never married	8,829	9,075	33.35
Currently married	14,529	16,189	59.49
Formerly/ever married	2,026	1,950	7.16
**Educational level**			
No education	8,816	10,026	36.84
Primary	9,957	11,705	43.00
Secondary	4,120	3,624	13.32
Higher	2,444	1,814	6.67
Don’t know	47	45	0.17
**Number of household members**			
1–3	5,888	5,468	20.09
4–6	11,629	13,028	47.87
>6	7,867	8,718	32.04
**Place of residence**			
Urban	8,146	5,560	20.43
Rural	17,238	21,654	79.57
**Region**			
Tigray	2,787	1,917	7.05
Afar	1,660	194	0.70
Amhara	3,249	7,257	26.92
Oromia	3,243	9,780	35.70
Somali	2,010	632	2.32
Benishangul	1,849	260	0.95
SNNPR	3,105	5,541	20.41
Gambella	1,763	75	0.27
Harari	1,257	51	0.19
Addis Ababa	2,750	1,366	4.95
Dire Dawa	1,711	141	0.51
**Wealth index**			
Poorest	6,009	4,381	16.10
Poorer	3,482	4,918	18.07
Middle	3,407	5,336	19.61
Richer	3,614	5,669	20.83
Richest	8,872	6,910	25.39
**Drinking water treatment**			
Yes	2,776	2,489	9.16
No	22,585	24,707	90.78
Don't know	23	18	0.06
**Cigarette smoking**			
Yes	773	405	1.72
No	24,506	26,721	98.28
**Types of toilet**			
Flush	1,743	844	3.10
Pit latrine	15,152	17,738	65.18
Open field	8,129	8,235	30.26
Other *	360	397	1.46
**Total**	**25,384**	**27,214**	**100%**

*Other: Composting toilet, bucket toilet, hanging toilet/latrine; SNNPR: Southern Nation and Nationality and Peoples Regions.

### Spatial analysis of underweight

#### Spatial distribution of underweight

**Fig 1:**
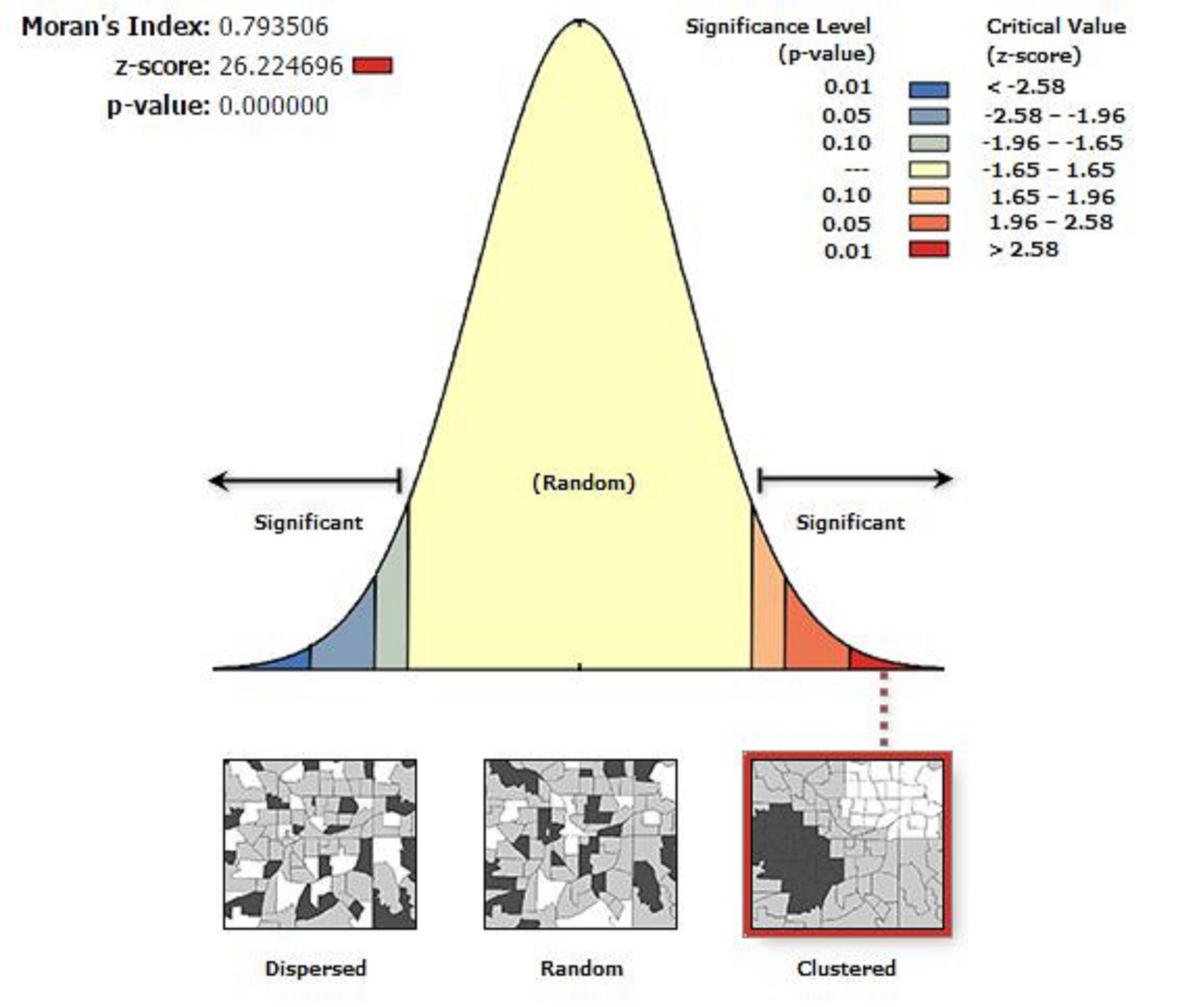
Spatial autocorrelation analysis of underweight among individuals aged 15–49 years in Ethiopia, 2016.

**Fig 2:**
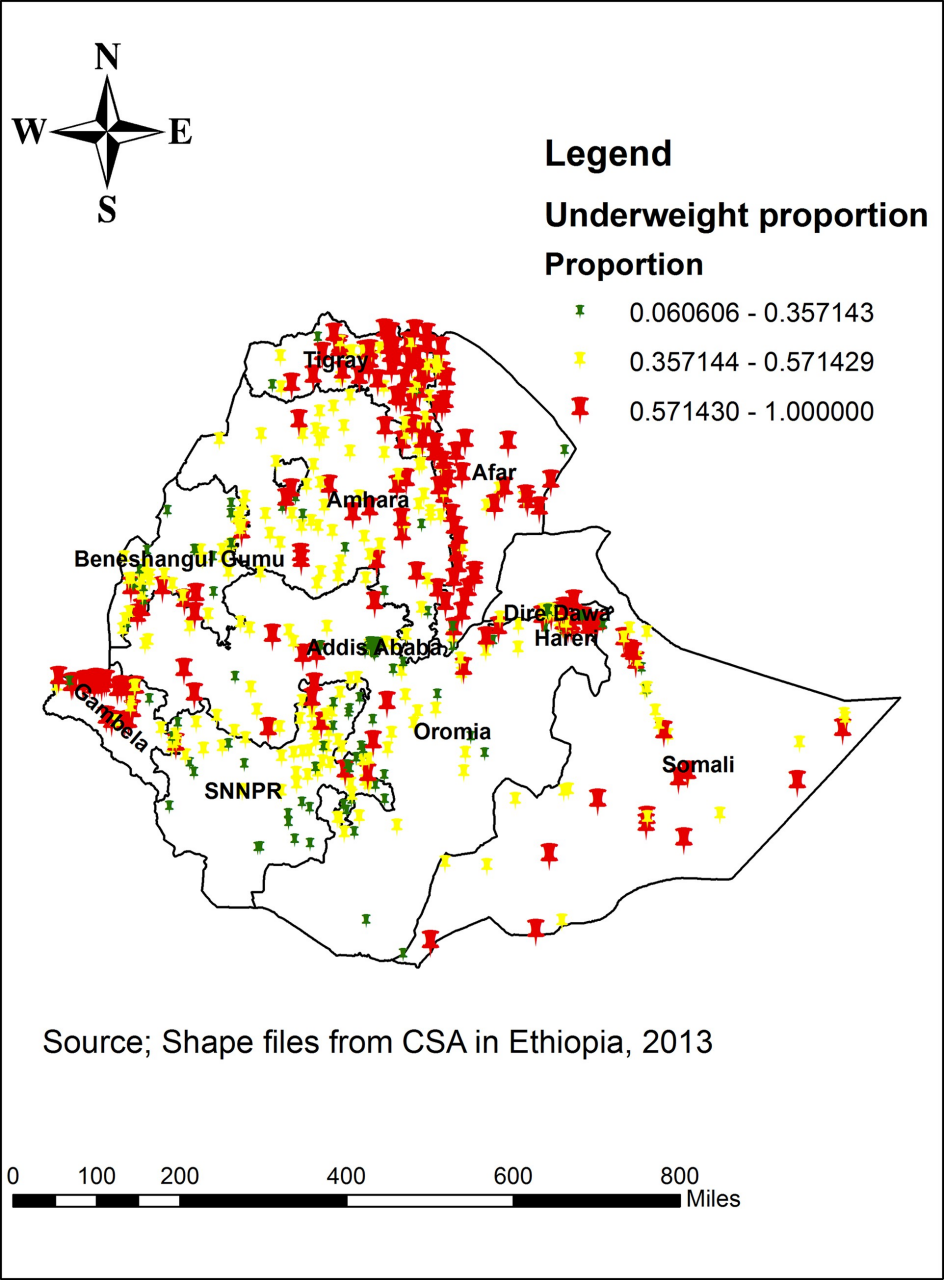
Spatial distribution of underweight across regions among individuals aged 15–49 years in Ethiopia, 2016.

The spatial distribution of underweight among individuals aged 15–49 years in Ethiopia was non-random (clustered) (Global Moran’s I  =  0.79, p value < 0.0001) (Fig 1). The highest proportions of underweight was located in the Tigray, Amhara, western and central part of Afar and Gambella regions, while low proportions of underweight was identified in the eastern part of SNNPR, western part of Oromia Harar, Dire Dawa and Addis Ababa (Fig 2).

#### Getis-Ord Gi* statistical analysis of underweight

**Fig 3:**
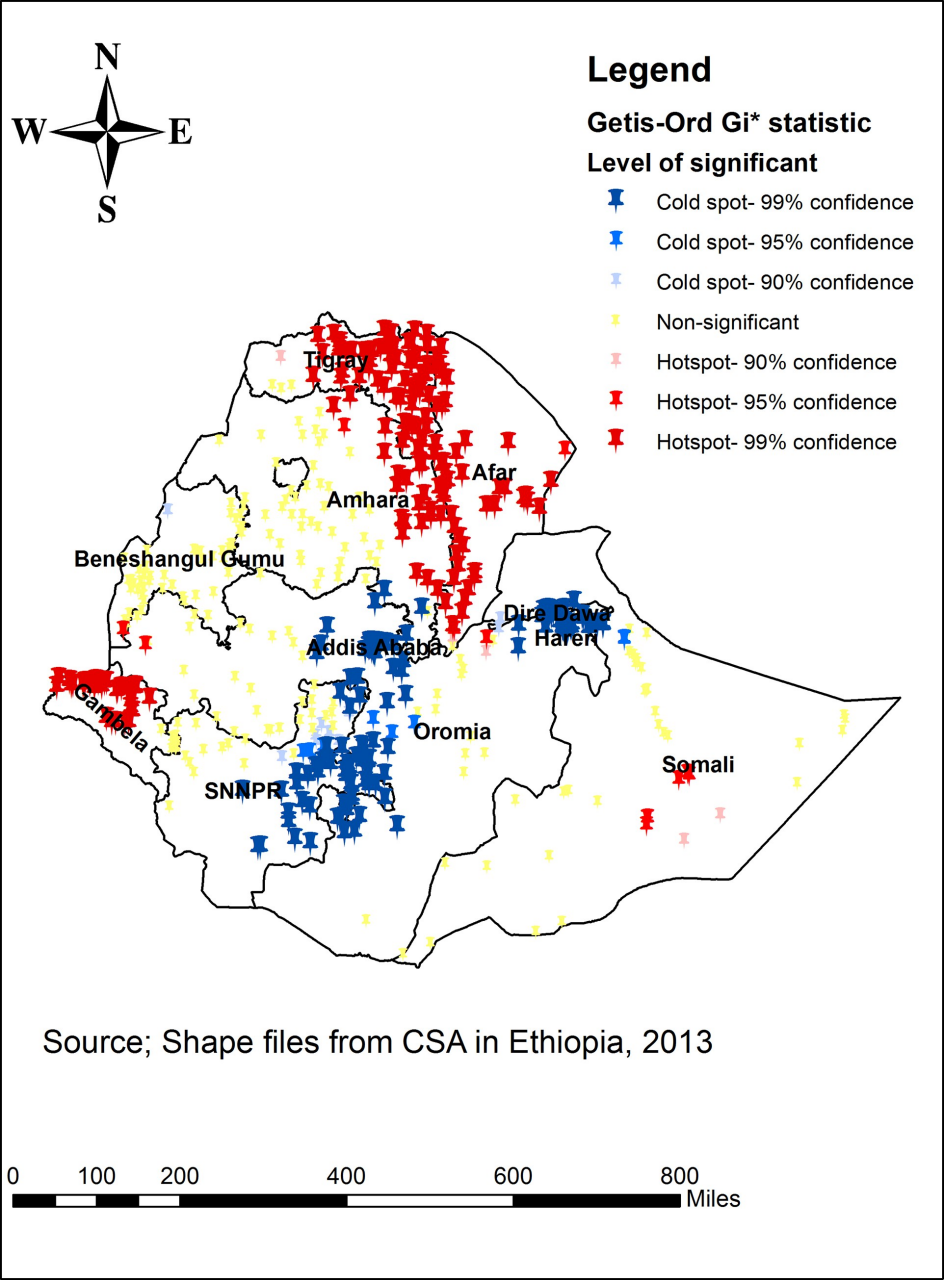
Hotspot and Cold spot underweight across regions among individuals aged 15–49 years in Ethiopia, 2016.

The Gettis-Ord Gi* statistical analysis indicated the hotspot and cold spot of underweight among individuals aged 15–49 years in Ethiopia. The red colors show the significant hotspot areas (higher cluster of underweight), which was located in Tigray, Gambella, eastern part of Amhara, western and central part of Afar regions. In contrast, the blue color indicates significant cold spot (lower cluster of underweight), identified in eastern part of SNNPR, western part of Oromia, Harar, Dire Dawa and Addis Ababa (Fig 3).

#### Spatial SaTScan analysis of underweight (Bernoulli based model)

**Fig 4:**
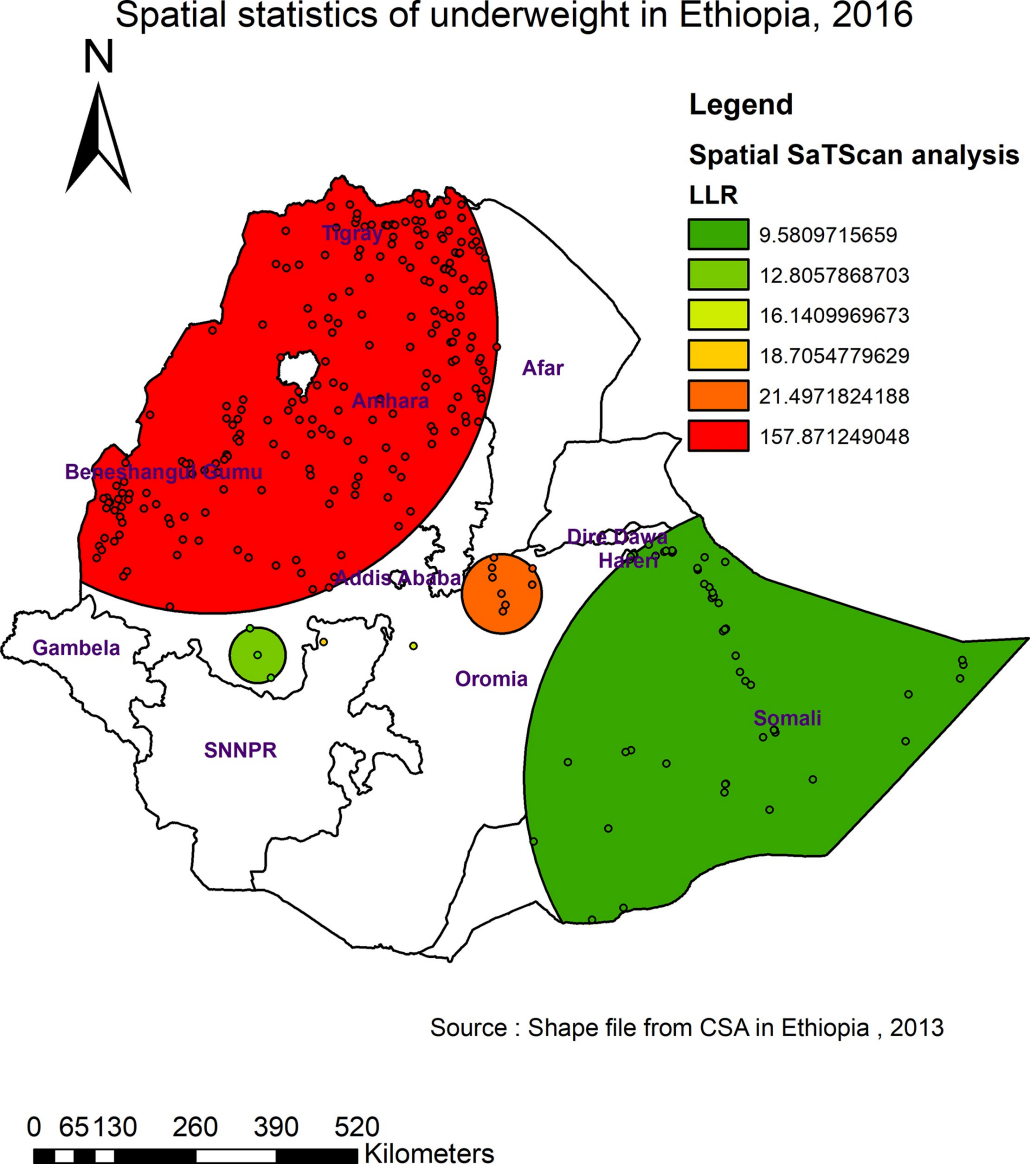
Primary and secondary clusters of underweight among individuals age 15–49 years in Ethiopia, 2016.

In the SaTScan analysis, a total of 279 significant clusters of underweight among individuals age 15–49 years in Ethiopia were identified (Fig 4). Among these, 216 were primary clusters. The primary clusters were found in Tigray, Amhara, Benishangul and western part of Afar at 12.579086 N, 36.033274 E with 457.42 km radius, a Relative Risk (RR) of 1.27, and Log-Likelihood Ratio (LRR) of 157.87, at p-value < 0.001. Individuals within the spatial window had 1.27 times higher chance of being underweight as compared to those individuals outside the spatial window (Table 3). The secondary clusters were located in the Gambella region.

**Table 3:** Significant spatial clusters with high rate underweight among individuals age 15–49 years in Ethiopia, 2016.

Cluster	Enumeration area (cluster) identified	Coordinate (radius)	Population	Case	RR	LLR	P-value
1	52, 279, 292, 259, 415, 602, 386, 504, 541, 296, 548, 638, 640, 256, 169, 361, 431, 515, 612, 516, 615, 73, 498, 312, 163, 158, 327, 253, 322, 382, 152, 533, 246, 109, 559, 512, 36, 167, 35, 258, 137, 364, 183, 80, 132, 150, 429, 3, 244, 403, 456, 628, 199, 24, 457, 184, 583, 188, 425, 494, 340, 375, 268, 120, 324, 474, 569, 98, 627, 285, 255, 320, 551, 409, 38, 65, 209, 181, 528, 78, 407, 88, 563, 584, 335, 595, 124, 66, 581, 545, 621, 542, 156, 206, 433, 508, 203, 531, 317, 597, 579, 636, 400, 6, 575, 482, 165, 416, 590, 176, 218, 81, 70, 229, 349, 17, 538, 350, 401, 300, 591, 84, 424, 161, 392, 355, 136, 374, 10, 481, 304, 478, 460, 45, 143, 294, 604, 430, 462, 160, 395, 461, 479, 449, 97, 354, 267, 237, 94, 550, 351, 200, 89, 510, 442, 616, 605, 275, 455, 280, 399, 220, 384, 193, 617, 226, 129, 79, 128, 410, 496, 598, 623, 643, 341, 421, 175, 404, 99, 249, 298, 488, 234, 517, 248, 18, 511, 196, 611, 332, 413, 572, 189, 344, 130, 345, 423, 117, 127, 241, 192, 172, 571, 485, 191, 362, 389, 235, 263, 103, 118, 254, 558, 585, 23, 544	(12.579086 N, 36.033274 E) / 457.42 km	11149	5692	1.27	157.87	< 0.001
2	122, 245, 529, 71, 49, 476, 51, 506	(8.757437 N, 40.299443 E) / 64.13 km	827	462	1.26	21.50	< 0.001

LLR: Likelihood ratio; RR: Relative risk.

### Factors associated with underweight

According to multivariable logistic regression analysis, gender, age, marital status, number of household members, place of residence, region, wealth index, drinking treated water, types of toilet and cigarette smoking were significantly associated with underweight among individuals aged 15–49 years in Ethiopia at p-value 0.05 (Table 4).

**Table 4:** Bi-variable and multivariable logistic regression analysis of underweight among individuals age 15–49 years in Ethiopia, 2016.

Variables	Underweight		Odds Ratio (95% CI)		P-value
	Yes	No	COR	AOR	
**Gender**					
Female	6,086	8,433	1	1	-
Male	6,125	6,570	1.25 (1.19, 1.31)	1.21 (1.15, 1.28)	<0.001**
**Age**					
15–19	3,071	2,981	1	1	
20–24	2,029	2,410	0.79 (0.73, 0.86)	0.87 (0.80, 0.96)	0.003*
25–29	1,957	2,840	0.67 (0.62, 0.73)	0.75 (0.68, 0.83)	<0.001**
30–34	1,548	2,355	0.63 (0.58, 0.68)	0.67 (0.60, 0.76)	<0.001**
35–39	1,485	1,924	0.71 (0.65, 0.78)	0.75 (0 .67, 0.85)	<0.001**
40–44	1,156	1,389	0.77 (0.70, 0.85)	0.78 (0.69, 0.88)	<0.001**
45–49	965	1,104	0.81 (0.73, 0.90)	0.82 (0.72, 0.94)	0.004*
**Marital status**					
Currently married	7,024	9,165	1	1	
Never married	4,327	4,748	1.06 (1.01, 1.12)	1.14 (1.05, 1.24)	0.002*
Formerly/ever married	860	1,090	0.93 (0.84, 1.02)	1.02 (0.92, 1.13)	0.069
**Educational level**					
No education	4,608	5,418	1	1	
Primary	5,549	6,156	0.90 (0 .85, 0.95)	1.01 (0.94, 1.08)	0.797
Secondary	1,436	2,188	0.66 (0.62, 0.72)	0.97 (0 .89, 1.07)	0.574
Higher	600	1,214	0.46 (0.42, 0.51)	0.91 (0 .81, 1.02)	0.116
Don’t know	18	27	0.57 (0 .29, 0.95)	0.65 (0.34, 1.26)	0.204
**Number of household members**					
1–3	2,270	3,198	1	1	
4–6	5,964	7,064	1.20 (1.13, 1.28)	1.07 (1.01, 1.15)	0.049*
>6	3,977	4,741	1.36 (1.27, 1.45)	1.06 (0.98, 1.14)	0.138
**Place of residence**					
Urban	1,779	3,781	1	1	
Rural	10,432	11,222	2.43 (2.30, 2.57)	1.32(1.18, 1.47)	<0.001**
**Region**					
Oromia	1,118	799	1	1	
Tigray	113	81	1.63(1.47, 1.81)	1.74 (1.56, 1.94)	<0.001**
Afar	3,522	3,735	1.94 (1.72, 2.19)	1.89 (1.65, 2.16)	<0.001**
Amhara	4,487	5,293	1.10 (1.00, 1.21)	1.08 (0.97, 1.19)	0.145
Somali	337	295	1.28 (1.14, 1.43)	1.22 (1.08, 1.38)	<0.001**
Benishangul	113	147	0.86 (0.77, 0.97)	0.84 (0.74, 0.94)	0.004*
SNNPR	2,075	3,466	0.68 (0.61, 0.75)	0.67 (0.64, 0.74)	<0.001**
Gambella	37	38	1.42 (1.26, 1.59)	1.62 (1.43, 1.83)	<0.001**
Harari	20	31	0.72 (0.63, 0.82)	1.04 (0 .90, 1.20)	0.590
Addis Ababa	334	1,032	0.37 (0.33, 0.41)	0.81 (0 .71, 0.93)	0.003*
Dire Dawa	55	86	0.70 (0.62, 0.79)	1.06 (0.94, 1.21)	0.278
**Wealth index**					
Poorest	2,220	2,161	1	1	
Poorer	2,478	2,440	0.76 (0.70, 0.83)	0.97 (0 .88, 1.07)	0.572
Middle	2,570	2,766	0.70 (0.64, 0.76)	0.93 (0.84, 1.03)	0.182
Richer	2,659	3,010	0.61 (0.56, 0.66)	0.85 (0.76, 0.94)	0.002*
Richest	2,284	4,626	0.31 (0.29, 0.33)	0.55 (0 .48, 0.63)	<0.001**
**Drinking water treatment**					
No	1,032	1,457	1	1	
Yes	11,170	13,537	0.77 (0.71, 0.83)	0.91 (0.83, 0.99)	0.031*
Don't know	9	9	0.90 (0.39, 2.05)	0.70 (0.30, 1.65)	0.415
**Types of toilet**					
Pit latrine	247	597	1	1	
Flush	7,526	10,212	0.58 (0.52, 0.65)	0.78 (0.69, 0.88)	<0.001**
Open field	4,262	3,973	2.03 (1.93, 2.15)	1.17 (1.08, 1.26)	<0.001**
Other	176	221	1.11 (0.90, 1.37)	1.05 (0.84, 1.31)	0.677
**Cigarette smoking**					
No	190	215	1	1	
Yes	11,970	14,751	1.42 (1.23, 1.64)	1.25 (1.07, 1.46)	0.005*
Hosmer-Lemeshow chi2 (10)	9.33(0.3150)				
Mean VIF (Max)	1.88(5.78)				

*: P ≤ 0.05

**: P ≤ 0.001; Other: Composting toilet, bucket toilet, hanging toilet/latrine; AOR: Adjusted Odd ratio; COR: Crude Odd Ratio; CI: Confidence interval; SNNPR: Southern Nation and Nationality and Peoples Regions; VIF: variance inflation factor.

Male individuals were 1.21 times more likely to be underweight than female individuals. The odds of being underweight were increased among individuals aged 20–59 years than individuals aged 15–19 years. Never married individuals were 1.14 times more likely to be underweight than married individuals. Persons who have lived with 4–6 household members were 1.07 times more likely to be underweight than persons who were living with 1–3 household members.

Regions were an important variable that showed significant association with underweight among individuals aged 15–49 years in Ethiopia. Persons residing in Tigray, Afar, Somali and Gambella were 1.74, 1.89, 1.22 and 1.62 times more likely to be underweight than a person residing in Oromia respectively, whereas the odds of underweight were decreased by 16 percent, 18 percent and 33 percent among individuals residing in Benishangul, Addis Ababa and SNNPR as compared with adult residing in Oromia respectively. Rural residents were 1.32 times more likely to be underweight than their counterpart.

Regarding wealth index, the probabilities of underweight were decreased by 15 percent and 45 percent among richer and richest individuals as compared with poorest individuals. Persons who smoke cigarette were 1.25 times more likely to be underweight than their counterpart. The odds of underweight were decreased by 9 percent among individuals who treat drinking water before use when compared with their counterpart.

Type of toilet was also another factor, which has a determinate effect on underweight in Ethiopia. The likelihoods of being underweight were decreased by 22 percent among person who use flush type of latrine than those who use a pit type of latrine. In contrast, persons who defecate in the open field were 1.17 times more likely to be underweight while compared with individuals who use a pit type of latrine.

## Discussion

According to the current study, the spatial distribution of underweight among individuals aged 15–49 years in Ethiopia was non-random. The significant hotspot areas with high rates of underweight were found in Tigray, Gambella, and eastern part of Amhara, western and central part of Afar region. In contrast, the significant cold spots with high rates of underweight were observed in the eastern part of SNNPR, western part of Oromia Harar, Dire Dawa and Addis Ababa. The possible explanation for this variation might be due to different socio-economical compositions of the study participants. Specifically, the majority of residents in Addis Ababa and Dire Dawa were rich in their wealth index, whereas majority dwellers of Afar and Gambella were poorer. Availability of natural resources may also be the reasons for this variation. These natural resources include water, land along with all vegetation and animal life. Regions like SNNPR and Oromia, Benishangul and Amhara have lands which are suitable for agriculture.

This study revealed that male individuals were more likely to be underweight than females. This finding is in line with a study conducted in Yemen [20]. The result shows that boys have less access to nutritious food and fall sick more frequently as compared to the girls. This similar finding might be explained by the fact that boys are more likely to be addicted to alcohol and cigarettes [21]. In line with studies conducted in Nepal [22], Indonesia [23] and India [24] the present study reported that the odds of being underweight were increased among adult age 15–19 years than adult age 20–59 years. Explanations for the increased odds of underweight during early adulthood may be related to food insecurity, fear of being fat, high physiologic need and eating disorder [25].

Marital status is a social factor which is of significance in health and mortality. According to the present study, the likelihoods of being underweight were decreased among married individuals than never married ones. A similar result has been found in Nepal [22]. This is because married people, especially men, are more likely to be healthier than unmarried men [26,27]. The current study also documented that a significant association between family size and underweight. Persons who have lived with 4–6 household members were more likely to be underweight than persons who were living with 1–3 household members. This finding may not be surprising because as the family size increases, keeping food security becomes a big issue, especially in poor families [28].

In agreement with studies conducted in Nepal [22] and Indonesia [23], the current study documented that rural residents were more likely to be underweight than urban dwellers. The possible explanations for such results might be rural residents are less likely to work in sedentary employment, use motorized transportation, eat diets high in processed grains and sugars [29,30]. Furthermore, because socioeconomic statuses are more likely to be lower among rural residents and this is associated with a lower BMI [31,32]. According to the present study, the odds of underweight were decreased among rich individuals than poor individuals, this is of course in congruent with another report [22]. This finding might be due to the fact that poverty intensifies the hazard of, and risks from, malnutrition. People who are poor are more likely to be suffering from distinct forms of malnutrition. Additionally, malnutrition increases health care costs, reduces productivity, and slows financial growth, which can perpetuate a cycle of poverty and ill-health [33].

The current study documented that persons who smoke cigarette were more likely to be underweight than their counterparts. This finding is consistent with other previous studies [34–36]. Such finding might be related to reasons like decrement in appetite and calorie intake, enhanced metabolism, and reduced fat accumulation [34]. This may be due to the effects of nicotine on brain’s regulation of appetite and energy expenditure [37,38].

The present study also reported that the significant association between types of latrine and being underweight. The odds of underweight were decreased among adult who treats drinking water before use when compared with their counterparts. Persons who defecate in the open field were more likely to be underweight while compared with persons who use a pit type of latrine. We included these two factors in the analysis because of their association with Feco-oral diseases. Especially those individuals who used untreated water and defecated in an open field are high risk of contracting fecal-oral diseases. Furthermore, these factors are signs of lower socio-economic status, which can determine the nutritional status of individuals.

The main strength of the present study was the use of nationally representative data. As a result, the findings of this study are generalizable to the target population of Ethiopia. Moreover, the simultaneous use of both ArcGIS and Sat Scan statistical tests enabled to find similar and statistically significant area with a high cluster of underweight (hot spot area).

This study has limitations that should be considered when interpreting the results. One of the limitations of the current study was, the location of data values was shifted up to 2 kilometers for urban and up to 5 kilometers for rural areas to ensure respondent confidentiality. Thus, this was the challenge to know the exact cases’ location. Since the current study used secondary data, some important variable like dietary intake, physical activity and sedentary lifestyle were not included in the analysis.

## Conclusion

There was a significant clustering of underweight among individuals age 15–49 years in Tigray, Gambella, and eastern part of Amhara, western and central part of Afar regions. The odds of being underweight were higher among males, individuals aged 15–19 years, never married, rural residents, poorest and cigarette smoker. Persons, who drank untreated water, defecate on open field and households with large family size were more likely to be underweight. Therefore, effective public health interventions like building safe and supportive environments for nutrition, providing socio-economic protection and nutrition-related education for poor and rural resident would be better to mitigate these situations and associated risk factors in hot spot areas. In addition, policymakers should strengthen and promote nutrition sensitive policies and activities in order to alleviate the underlying and basic causes of underweight.

## Supporting information

S1 Datasets (XLS)

## Abbreviations

AOR: Adjusted Odds Ratio
BMI: Body mass index
CI: Confidence Interval
CSA: Central Statistics Agency
DHS: Demography heath survey
EAs: Enumeration Areas
EDHS: Ethiopian Demographic and Health Survey
SNNPR: Southern Nation and Nationality and Peoples Regions
VIF: variance inflation factor
WHO: World Health Organization
